# Antibiotic Resistance Profile of Uropathogenic Bacteria in Diabetic Patients at the Bafoussam Regional Hospital, West Cameroon Region

**DOI:** 10.7759/cureus.9345

**Published:** 2020-07-22

**Authors:** Arsene Tejiogni Signing, Wiliane Jean Takougoum Marbou, Veronique Penlap Beng, Victor Kuete

**Affiliations:** 1 Biochemistry, University of Dschang, Dschang, CMR; 2 Biochemistry, University of Yaoundé 1, Yaoundé, CMR

**Keywords:** urinary tract infections, bacteria resistance, diabetes

## Abstract

Background

Urinary tract infections constitute a major public health concern. The aim of the study is to look into the antibiotic sensitivity profile of uropathogenic bacteria among diabetic individuals in the Diabetology Unit of the Bafoussam Regional Hospital, West Cameroon.

Methods

A cross-sectional study was carried out in Bafoussam Regional Hospital. Urine was collected in a sterile jar previously labelled. The microorganisms were isolated on agar medium and their final identification was carried out on the API20E gallery. The antibiogram was performed using agar diffusion methods.

Results

*Escherichia coli* (25.30%) and *Staphylococcus aureus *(19.27%) were the most noticed species in the diabetic patients, whereas *Escherichia coli* (32.00%) and *Klebsiella pneumoniae* (22.00%) were the most noticed in non-diabetic patients. There was a significant association between antibiotic resistance and diabetic status (for ceftriaxone: X^2 ^= 23.78 and P-value < 0.001; for cefixime: X^2 ^= 19.31 and P-value < 0.001; for ceftazidime: X^2 ^= 9.45 and P-value = 0.008; for cefotaxime: X^2 ^= 10.97 and P-value = 0.004; for cefepime: X^2 ^= 27.93 and P-value < 0.001; and for ciprofloxacin: X^2 ^= 11.13 and P-value = 0.003). Multidrug resistance rate against some bacterial species were higher in diabetic patients (62.50% for *Escherichia coli*, 63.16% for *Klebsiella pneumoniae*, and 78.57% for *Staphylococcus aureus*) compared with non-diabetic patients (37.50% for *Escherichia coli*, 36.84% for *Klebsiella pneumoniae*, and 21.43% for *Staphylococcus aureus*).

Conclusions

This study revealed that there is an association between antibiotic resistance and diabetic status. Research and interventions must be focused on the elderly diabetic population in order to fight against the occurrence of drug-resistant uropathogenes.

## Introduction

Today, infectious diseases constitute a real global health problem due to the evolving power of microorganisms, from social and technological attitudes associated with negligence, and the uncontrolled use of antibiotics [1]. They continue to emerge in high frequency and pose multiple problems on a scale that no continent has been spared. In Cameroon, these infections include diseases such as diarrhea, cholera, and typhoid fever, as well as respiratory diseases [2].

Contrary to some infectious diseases that have an acute action, other diseases described as chronic invade the world in this era and also represent a major public health problem since they are responsible for a large number of deaths and are most often asymptomatic. One of the most alarming of these is diabetes. Indeed, diabetes mellitus is a metabolic disorder characterized by chronic hyperglycemia with an excessively high blood sugar level corresponding to a fasting blood sugar level greater than 1.26 g/L (7 mmol/L) twice linked deficiency in either insulin secretion, insulin action, or both [3].

Diabetes like other chronic diseases (cardiovascular diseases, cirrhosis, cardiac, respiratory or renal insufficiency, etc.) decreases the resistance of the body by altering phagocytic activity, thereby weakening immunity and thus making the body vulnerable to many infections such as urinary tract infections [4].

Urinary tract infections would be more frequent in diabetic patients according to the study carried out by Kamoun et al. [5]; they are at the origin of the increase in bacterial adhesion, decreased cytokine secretion, and the presence of glycosuria. The study by Mehvish and Betty assessing the prevalence of urinary tract infections in diabetic patients showed that the prevalence of urinary tract infections in poor socioeconomic countries is 56.4% for diabetic patients and 43.6% for non-diabetic patients. In countries with high socioeconomic status, this prevalence is 51.6% for diabetic patients and 48.4% for non-diabetic patients [6]. These results from their studies show that urinary tract infections would occur more on the diabetic population as opposed to the non-diabetic population.

Indeed, urinary tract infections are frequent both in hospitals and in community settings. In recent years, there has been an increase in the incidence of resistance of germs responsible for urinary tract infections to antibiotics; this was due to the emergence production of *Enterobacteriaceae* called extended-spectrum beta-lactamase [7].

Following the phenomenon of bacterial multidrug resistance, the sensitivity of these bacteria to antibiotics decreases considerably in diabetic patients, as seen in a study carried out at the Gondar University Hospital in Ethiopia on the prevalence of multidrug resistance of these bacteria to antibiotics that found that prevalence of bacterial multidrug resistance in urinary tract infections in diabetic patients was 59.8% [8]. Indeed, the resistance of bacteria to antibiotics in the general population and in diabetics in particular constitutes a public health problem today [9]. The multidrug resistance of uropathogenic bacteria in diabetic patients shows that diabetes is involved in a modification of the bacterial epidemiology of urinary tract infections and leads to greater resistance to the antibiotics used to treat them.

Studies on this relationship are very rare at the international level and practically non-existent in the national territory; therefore, the scope of this work is aimed at identifying the different germs involved in urinary tract infections in individuals of the Diabetology Unit of the Bafoussam Regional Hospital, West Cameroon, and to assess their behavior toward available antibiotics.

## Materials and methods

Study setting

The study was conducted in Bafoussam Regional Hospital. The hospital serves as a referral center for 20 hospitals in the western region districts.

Study design, participants, and sample size

A cross-sectional study was conducted from August 1, 2018, to May 29, 2019. The sample population included diabetes confirmed individuals at the Diabetology Unit and the non-diabetics individuals who came to consult at the diabetes unit of Bafoussam Regional Hospital and for whom urine culture had been prescribed. The sample size was calculated using the single population proportion formula by considering the sample proportion as 5.8% prevalence of diabetes [10], 0.03 desired precision, 95% confidence interval (CI), and a design effect of 2. Thus, the minimum sample size (n) calculated was found to be 468. We, therefore, included 455 diabetic patients (41 type I diabetes and 414 type II diabetes) and 50 non-diabetic patients for a total of 505 participants. Type I diabetes and type II diabetes were considered as the inclusion criteria.

Ethical considerations

Ethical clearance for this study was obtained from the Ethics Review and Consultancy Committee, Cameroon Bioethics Initiative (CAMBIN). We obtained a research certificate from the University of Dschang as well as a research authorization from the Bafoussam Regional Hospital. All the participants were duly informed of the study's goals, procedures, potential harm and benefits, and cost, as well as the finality of the study. Each patient signed an informed consent form, thereby agreeing to participate in the study. Subsequently, a questionnaire was submitted to them and the collection of samples was carried out following scientific and ethical standards. All results were coded and kept confidential.

Non-inclusion criteria 

Pregnant women, tuberculosis patients, and HIV-positive patients were excluded from this study to avoid the possible impact on anthropometric and laboratory parameters.

Data collection

During our study, patients had to fill in a data sheet concerning their identification (sex, age, hospitalization, surgery, taking antibiotic and treatment, and date of last urinary tract infection) to be able to estimate the risk factors of the infection.

Biochemical measurements

Plasma glucose (after an overnight fasting of eight or more hours) was determined using the glucose meter Accu-Chek Active system (Roche Diabetes Care, Basel, Switzerland) [11]. Fasting capillary blood samples were collected three times (for three consecutive hours) from a single study participant, and glucose measurement was carried out within fractions of seconds after sample collection. Then, their average was taken for analysis. The diagnosis of DM was based on the American Diabetes Association diabetes mellitus classification criteria with fasting blood glucose (FPG) of ≥126 mg/dL being considered as positive for diabetes and FPG of less than 61 mg/dL to <110 mg/dL being considered normoglycemic [12]. An FPG level of >126 mg/dL or a casual plasma glucose level of >200 mg/dL meets the threshold for the diagnosis of diabetes.

Urine collection

A small amount of urine (about 25 mL) was collected in a sterile jar previously labelled. All samples were taken before any antibiotic therapy in all patients. The samples were preceded by hand hygiene and a toilet in the urethral or vulvar region. The method of collection used was “midstream urine”. Concerning the patients on a catheter, disinfection of the specific site of the probe device before the collection of urine by puncture had been made.

Bacteriological study

The collected urine was stored at room temperature and analyzed within a maximum of two hours after reception. Ten microliters of each homogenized sample were inoculated on the cystine lactose electrolyte deficient (CLED) and eosin methylene blue (EMB) agars to isolate the Gram-negative bacilli and on the Chapman (mannitol salt) agar to isolate the Gram-positive cocci. The incubation was carried out at 37°C for 24 hours. After isolation and purification, the bacteria were put on clean slides, and Gram stain was performed and then observed under the microscope at the 100x objective. Gram-negative bacilli (*Enterobacteriaceae*) and Gram-positive cocci (*Staphylococcus*) were observed. The final identification of* Enterobacteriaceae* was carried out on the API20E (bioMérieux, Lyon, France) gallery and that of staphylococci was carried out with the help of additional tests (catalase and coagulase).

Antimicrobial susceptibility testing

Isolated bacteria were identified and submitted to an antibiogram test to determine their antibiotic sensitivity profile. The antibiogram was performed on the Mueller-Hinton medium, applying the diffusion method in agar medium, and the interpretation was made according to the standards of the Antibiogram Committee of the French Society for Microbiology (AC-FSM) [13]. Each germ was tested by antibiotics that are specific to it according to the AC-FSM standards.

The sensitivity of the isolated bacteria was assessed. We tested the sensitivity of the Gram-negative bacilli obtained to 17 antibiotics: sparfloxacin (SPX, 5 µg), imipenem (IPM, 10 µg), levofloxacin (LEV, 5 µg), ceftazidime (CAZ, 30 µg), amikacin (AK, 30 µg), cefotaxime (CTX, 5 µg), cefixime (CFM, 5 µg), netilmicin (NET, 10 µg), ciprofloxacin (CIP, 30 µg), ceftriaxone (CRO, 30 µg), gentamicin (CN, 10 µg), chloramphenicol (CHL, 30 µg), amoxicillin and clavulanic acid (AMC, 10 µg), amoxicillin (AMX, 30 µg), tobramycin (TOB, 10 µg), ampicillin (AMP, 10 µg), and cefepime (CFP, 5 µg) (Becton Dickinson and Company, Sparks, MD, USA).

However, we tested the sensitivity of Gram-positive cocci to nine antibiotics: oxacillin (OX, 5 µg), streptomycin (STR, 10 µg), vancomycin (VAN, 30 µg), doxycycline (DOX, 30 µg), penicillin (PEN, 10 IU), SPX (5 µg), minocycline (MI, 30 µg), IPM (10 µg), LEV (5 µg) (Becton Dickinson and Company).

Data analysis

To examine the association of diabetes status with antibiotic-resistance, we used the chi-square (X^2^) test for categorical variables. P-values < 0.05 were significant.

## Results

Descriptive statistics

Of the total 505 participants, 455 were diabetic patients and 50 were non-diabetic patients. The average age of the total study participants was 54.75 ± 15.78 years and that of diabetic patients was 56.94 ± 14.33 years. In non-diabetic patients, the average age was 34.60 ± 14.35 years. There was a significant difference between the average ages of diabetics compared to the average age of non-diabetic patients (P-value < 0.05). From 455 diabetic patients, we obtained 372 negative urine culture (81.75%) and 83 positive cultures, giving a frequency of urinary tract infection of 18.24%; 73.50% (n = 61) were women and 26.50% (n = 22) were men. In non-diabetic patients, 54% (n = 27) were women and 46% (n = 23) were men.

Bacteria isolated from the total population

Urine culture allows the diagnosis of a urinary tract infection (cystitis, prostatitis, pyelonephritis) in adults, children, and infants by identifying the nature of the germ (bacteria that cause more than 90%, parasite, or fungus) causing this infection. The bacterial identification of positive urine samples allowed us to determine the different kinds of bacteria implicated in these infections and to calculate their relative frequency.

In the general population, the species *Escherichia coli*, *Klebsiella pneumoniae*, and *Staphylococcus aureus* were the most represented with the respective percentages of 27.82%, 19.55%, and 17.29%. *Serratia liquefaciens*, *Proteus penneri*, *Escherichia fergusonii*, *Citrobacter freundii*, *Aeromonas hydrophila* 2, and *Staphylococcus epidermidis* were the least represented species (0.75% for each) (Table 1).

**Table 1:** Frequency of isolated bacteria in the total population LCL, lower control limit; UCL, upper control limit

Identified bacteria	Frequency	Percent	Cumulative percent	Exact 95% LCL	Exact 95% UCL
Escherichia coli	37	27.82%	37.59%	20.40%	36.25%
Klebsiella pneumoniae	26	19.55%	57.14%	13.19%	27.32%
Staphylococcus aureus	23	17.29%	74.44%	11.29%	24.81%
Aeromonas hydrophila 1	13	9.77%	9.77%	5.31%	16.13%
Enterobacter cloacae	10	7.52%	83.46%	3.66%	13.39%
Proteus mirabilis	8	6.02%	96.24%	2.63%	11.51%
Klebsiella oxytoca	4	3.01%	86.47%	0.83%	7.52%
Serratia marcescens	4	3.01%	89.47%	0.83%	7.52%
Serratia odorifera	2	1.50%	75.94%	0.18%	5.33%
Serratia liquefaciens	1	0.75%	90.23%	0.02%	4.12%
Proteus penneri	1	0.75%	96.99%	0.02%	4.12%
Escherichia fergusonii	1	0.75%	97.74%	0.02%	4.12%
Citrobacter freundii	1	0.75%	98.50%	0.02%	4.12%
Aeromonas hydrophila 2	1	0.75%	99.25%	0.02%	4.12%
Staphylococcus epidermidis	1	0.75%	100.00%	0.02%	4.12%
Total	133	100,00%	100,00%		

Bacteria isolated according to diabetic status

In the diabetic population, the most represented species were *Escherichia coli* (25.30%), *Staphylococcus aureus* (19.27%), and *Klebsiella pneumoniae* (18.07%). *Escherichia fergusonii*, *Citrobacter freundii*, *Aeromonas hydrophila* 2, and *Staphylococcus epidermidis* were absent (0.00%) (Table 2).

**Table 2:** Frequency of bacteria isolated according to diabetic status

Identified bacteria	Diabetic patients (%)	Non-diabetic patients (%)	Total
Escherichia coli	21 (25.30)	16 (32.00)	37
Klebsiella pneumoniae	15 (18.07)	11 (22.00)	26
Staphylococcus aureus	16 (19.27)	7 (14.00)	23
Aeromonas hydrophila 1	9 (10.84)	4 (8.00)	13
Enterobacter cloacae	8 (9.63)	2 (4.00)	10
Proteus mirabilis	5 (6.02)	3 (6.00)	8
Klebsiella oxytoca	2 (2.41)	2 (4.00)	4
Serratia marcescens	4 (4.82)	0 (0.00)	4
Serratia odorifera	1 (1.20)	1 (2.00)	2
Serratia liquefaciens	1 (1.20)	0 (0.00)	1
Proteus penneri	1 (1.20)	0 (0.00)	1
Escherichia fergusonii	0 (0.00)	1 (2.00)	1
Citrobacter freundii	0 (0.00)	1 (2.00)	1
Aeromonas hydrophila 2	0 (0.00)	1 (2.00)	1
Staphylococcus epidermidis	0 (0.00)	1 (2.00)	1
Total	83 (100%)	50 (100%)	133

For non-diabetics, *Escherichia coli *(32.00%), *Klebsiella pneumoniae* (22.00%), and *Staphylococcus aureus* (14.00%) were also the most represented species. On the other hand, the absent species were *Serratia marcescens*, *Serratia liquefaciens,* and *Proteus penneri *(0.00%). There was no significant association between the identification of bacteria and diabetic status (X^2^ = 13.47 and P-value = 0.489) (Table 2).

Frequency of antibiotic resistance according to diabetic status

From Table 3, which shows the frequency of resistance to tested antibiotics based on diabetic status, it appears that the resistance is very high for AMP and AMX antibiotics in diabetics (87.04% and 75.68%, respectively) compared to non-diabetics (12.96% and 24.32%, respectively). There was a significant association between resistance to these two antibiotics and diabetic status (for AMP: X2 = 30.53 and P-value < 0.001; for AMX: X2 = 22.39 and P-value < 0.001).

**Table 3:** Frequency of antibiotic resistance of bacteria isolated from the total population I, intermediate; R, resistant; S, sensitive; AMP, ampicillin; AMX, amoxicillin; IMP, imipenem; CRO, ceftriaxone; CFM, cefixime; CAZ, ceftazidime; CTX, cefotaxime; CFP, cefepime; CIP, ciprofloxacin; OX, oxacillin; PEN, penicillin; SPX, sparfloxacin; LEV, levofloxacin; CN, gentamicin; NET, netilmicin; AK, amikacin; TOB, tobramycin; STR, streptomycin; CHL, chloramphenicol; DOX, doxycycline; MI, minocycline; VAN, vancomycin; AMC, amoxicillin and clavulanic acid.

Antibiotics	Sensitivity profile	Diabetic patients (%)	Non-diabetic patients (%)	X^2^	P-value
AMP	I (20)	9 (45.00)	11 (55.00)	30.53	<0.001
	R (54)	47 (87.04)	7 (12.96)		
	S (35)	11 (31.42)	24 (68.57)		
AMX	I (19)	4 (21.05)	15(78.95)	22.39	<0.001
	R (74)	56 (75.68)	18 (24.32)		
	S (15)	7 (46.66)	8 (53.33)		
IMP	I (22)	11 (50.00)	11 (50.00)	2.20	0.332
	R (21)	15 (71.42)	6 (28.57)		
	S (90)	57 (63.33)	33 (36.66)		
CRO	I (25)	9 (36.00)	16 (64.00)	23.78	<0.001
	R (47)	41 (87.23)	6 (12.77)		
	S (37)	17 (45.95)	20 (54.05)		
CFM	I (18)	4 (22.22)	14 (77.77)	19.31	<0.001
	R (84)	61 (72.62)	23 (27.38)		
	S (7)	2 (28.57)	5 (71.43)		
CAZ	I (25)	9 (36.00)	16 (64.00)	9.45	0.008
	R (74)	50 (67.57)	24 (32.43)		
	S (10)	8 (80.00)	2 (20.00)		
CTX	I (30)	12 (40.00)	18 (60.00)	10.97	0.004
	R (57)	43 (75.43)	14 (24.56)		
	S (22)	12 (54.54)	10 (45.45)		
CFP	I (38)	16 (42.10)	22 (57.90)	27.93	<0.001
	R (57)	48 (84.21)	9 (15.79)		
	S (14)	3 (21.42)	11 (78.57)		
CIP	I (15)	7 (46.66)	8 (53.33)	11.13	0.003
	R (45)	36 (80.00)	9 (20.00)		
	S (49)	24 (48.98)	25 (51.02)		
DOX	I (1)	0 (0.00)	1 (100.00)	3.48	0.175
	R (17)	13 (76.47)	4 (23.53)		
	S (6)	3 (50.00)	3 (50.00)		
PEN	I (16)	9 (56.25)	7 (43.75)	2.42	0.297
	R (7)	6 (85.71)	1 (14.90)		
	S (1)	1 (100.00)	0 (0.00)		
SPX	I (27)	11 (40.74)	16 (59.26)	8.14	0.017
	R (40)	30 (75.00)	10 (25.00)		
	S (66)	42 (63.63)	24 (36.36)		
LEV	I (17)	7 (41.17)	10 (58.82)	7.95	0.018
	R (73)	53 (72.60)	20 (27.40)		
	S (43)	23 (53.49)	20 (46.51)		
CN	I (13)	4 (30.77)	9 (69.23)	20.74	<0.001
	R (50)	42 (84.00)	8 (16.00)		
	S (46)	21 (45.65)	25 (54.34)		
NET	I (18)	8 (44.44)	10 (55.55)	3.56	0.168
	R (49)	34 (69.39)	15 (30.61)		
	S (42)	25 (59.52)	17 (40.78)		
AK	I (17)	9 (52.94)	8 (47.05)	1.17	0.556
	R (36)	21 (58.33)	15 (41.66)		
	S (56)	37 (66.07)	19 (33.92)		
TOB	I (23)	10 (43.48)	13 (56.52)	4.65	0.097
	R (54)	34 (62.96)	20 (37.03)		
	S (32)	23 (71.87)	9 (28.12)		
STR	I (21	0 (0.00)	1 (100.00)	2.53	0.281
	R (9)	7 (77.77)	2 (22.22)		
	S (14)	9 (64.29)	5 (35.71)		
CHL	I (15)	8 (53.33)	7 (46.66)	12.51	0.001
	R (68)	50 (73.53)	18 (26.47)		
	S (26)	9 (34.61)	17 (65.38)		
DOX	I (3)	1 (33.33)	2 (66.66)	4.80	0.090
	R (15)	9 (60.00)	6 (40.00)		
	S (6)	6 (100.00)	0 (0.00)		
MI	I (5)	2 (40.00)	3 (60.00)	3.28	0.193
	R (10)	7 (70.00)	3 (30.00)		
	S (8)	7 (87.50)	1 (12.50)		
VAN	I (2)	2 (100.00)	0 (0.00)	1.20	0.548
	R (12)	8 (66.66)	4 (33.33)		
	S (10)	6 (60.00)	4 (40.00)		
AMC	I (16)	3 (18.75)	13 (81.25)	21.57	<0.001
	R (83)	61 (73.49)	22 (26.51)		
	S (10)	3 (30.00)	7 (70.00)		

Also, the antibiotics CRO, CFM, CAZ, CTX, CFP, and CIP have a very high resistance in diabetics (87.23%, 72.62%, 67.57%, 75.43%, 84.21%, and 80.00%, respectively) compared to non-diabetics (12.77%, 27.38%, 32.43%, 24.56%, 15.79%, and 20.00%, respectively). There was a significant association between resistance to these antibiotics and diabetic status (for CRO: X^2^ = 23.78 and P-value < 0.001; for CFM: X^2^ = 19.31 and P-value < 0.001; for CAZ: X^2^ = 9.45 and P-value = 0.008; for CTX: X^2^ = 10.97 and P-value = 0.004; for CFP: X^2^ = 27.93 and P-value < 0.001 and for CIP: X^2^ = 11.13; and P-value = 0.003) (Table 3).

We also noted a significant association between antibiotic resistance and diabetic status (for SPX: X^2^ = 8.14 and P-value = 0.017; for LEV: X^2^ = 7.95 and P-value = 0.018; for CN: X^2^ = 20.74 and P-value < 0.001; for CHL: X^2^ = 12.51 and P-value = 0.001; and for AMC: X^2^ = 21.57 and P-value < 0.001). There is also a high resistance to these antibiotics in diabetics (75.00%, 72.60%, 84.00%, 73.23%, and 73.49%, respectively) compared to non-diabetics (52.00%, 27.40%, 16.00%, 26.47%, and 26.51%, respectively) (Table 3).

Multidrug resistance of bacteria identified according to diabetic status

The most multidrug-resistant bacteria species were *Escherichia coli*, *Klebsiella pneumoniae*, and *Staphylococcus aureus*. Multidrug resistance rates against these bacterial species were higher in diabetic patients (62.50% for *Escherichia coli*, 63.16% for *Klebsiella pneumoniae*, and 78.57% for *Staphylococcus aureus*) compared to non-diabetics (37.50% for *Escherichia coli*, 36.84% for *Klebsiella pneumoniae*, and 21.43% for *Staphylococcus aureus*). There was no significant difference between multidrug resistance to tested antibiotics and diabetic status (Table 4).

**Table 4:** Frequency of multidrug resistance to bacteria identified in the total population Na, non-applicable

Identified bacteria	Frequency of multidrug resistance of identified bacteria	Diabetic patients (%)	Non-diabetic patients (%)	X^2^	P-value
Escherichia coli	24	15 (62.50)	9 (37.50)	0.91	0.337
Klebsiella pneumoniae	19	12 (63.16)	7 (36.84)	0.86	0.352
Staphylococcus aureus	14	11 (78.57)	3 (21.43)	1.37	0.241
Aeromonas hydrophila 1	10	8 (80.00%)	2 (20.00%)	2.35	0.124
Enterobacter cloacae	8	7 (87.50)	1 (12.50)	1.40	0.235
Proteus mirabilis	7	4 (57.14)	3 (42.86)	0.68	0.407
Serratia marcescens	4	4 (100.00)	0 (0.00)	Na	Na
Klebsiella oxytoca	3	2 (66.67%)	1 (33.33%)	1.33	0.248
Serratia odorifera	1	1 (100.00)	0 (0.00)	2.00	0.157
Serratia liquefaciens	1	1 (100.00)	0 (0.00)	Na	Na
Proteus penneri	1	1 (100.00)	0 (0.00)	Na	Na
Citrobacter freundii	1	0 (0.00)	1 (100.00)	Na	Na
Aeromonas hydrophila 2	1	0 (0.00)	1 (100.00)	Na	Na

## Discussion

Urinary tract infections remain one of the most common problems that clinicians are facing. They occupy a special place in nephrological pathology by their frequency in both sexes and all ages. It is a serious infection due to its repercussions on the activities of the patients as well as its recurrences and its serious consequences. This study aims to identify the various germs involved in urinary tract infections in individuals in the Diabetology Unit of the Bafoussam Regional Hospital and to assess their behavior toward available antibiotics.

Thus, for our study with a sample of 455 diabetic patients, we obtained 372 negative urine cultures (81.75%) and 83 positive cultures, giving a frequency of urinary tract infection of 18.24%. This positivity rate is slightly higher than that obtained in subsequent studies by Guermazi-Toumi al. in Tunisia [14] and Ejaz et al. in Pakistan [15] who obtained positive urine, and culture rates of 16.5% and 6.3%, respectively. This situation is justified by the fact that urinary tract infection is one of the most frequent community bacterial infections [16]. The negative urine culture rate represents more than three-quarters of all the examinations carried out in our study. This can be explained by the fact that these patients are subjected to self-medication without medical consultation, which contributes to hide the pathogenic bacterial flora and hinders its multiplication on the culture media in the laboratory [17].

The high frequency of urinary tract infections in women compared to that in men is consistent with that reported in the literature review [17]. In diabetic patients, 73.50% were women, whereas 26.50% were men. In non-diabetic patients, 54% were women and 46% were men. This is explained by the fact that urinary tract infection in women can be interpreted by several factors related to the anatomical and physiological nature of their urinary tract since the length of their urethra is much reduced (4 cm). Also, hormonal and physiological changes can promote the occurrence of these infections [17]. Man is relatively more protected because of the anatomical structure of his urinary system. Fecal contamination is then reduced since there is a distance between his anus and his urinary meat [17].

Due to the state of immunosuppression and age, urinary tract infection is common in elderly patients and it is aggravated by the frequency of severe and complicated forms of infection. Thus, among the diabetics, 42 (50.60%) were aged 60 years and above and 32 (38.55%) were aged between 40 and 59 years. This high prevalence can be explained by several factors such as the aging of the urinary excretion system or a reduction of the muscular tone of the bladder walls, which involves a bladder stasis responsible for the proliferation of germs by reducing the urinary excretion rate [18]. Between the ages of 20 and 39 years, there is an intense sexual phase in men. This could explain the fact that it is the age group where urinary tract infection is higher in non-diabetics.

The ascending pathophysiology of urinary tract infection and the strong colonization of the perineum by *Enterobacteriaceae* of digestive origin, and in particular *Escherichia coli*, associated with specific factors of uropathogenicity such as bacterial adhesins capable of binding to the urinary epithelium [19] explain that the species *Escherichia coli* dominate the epidemiological profile. Gram-positive bacteria have an adhesion factor, which is lipoteichoic acid, which may explain why the species *Staphylococcus aureus* is represented with a rate of 17.29% [19]. The rate obtained for *Klebsiella pneumoniae* species (19.55%) is closed to that found by other studies, thus testifying that it is a species frequently encountered in urinary tract infections on diabetics [19].

In our study, the resistance of germs to beta-lactamines was very high. Kibret and Abera also found in their study high rates of resistance to antibiotics such as AMX and cephalotin [19]. Likewise, Ejaz et al. obtained high resistance for CFM and TOB antibiotics [15]. These resistance rates were 90.6% and 72% respectively. We also noticed that bacteria were also much more resistant to antibiotics of the aminoglycoside and tetracycline class, which is in line with the work carried out by Kibret and Abera [19]. Concerning phenicolates, we found resistance rates of 73.53%; these resistance rates are very high compared to those reported by other authors in the literature review [19]. These differences in the sensitivity of germs to antibiotics, which vary widely from one study to another, could be explained by the differences in bacterial ecologies and the conditions of antibiotic use, which remain very variable. The very high resistance rates against beta-lactamines and quinolones found in our work could be explained by the known practice of self-medication, the illicit sale and use of drugs, and the proliferation of clandestine health care units managed by unqualified health personnel who routinely prescribe antibiotics in doses and durations that are non-compliant. This could also explain the fact that the bacterial species *Escherichia coli*, *Klebsiella pneumoniae*, and *Staphylococcus aureus* are most multidrug-resistant.

The results of this study are of great importance to public health because the antibiotic resistance of uropathogens is not systematically sought in people suffering from diabetes. The results of this study would certainly contribute to the awareness and prevention of diabetes in people suffering from urinary tract infections. However, several limitations could be considered. First, the cross-sectional design limits the ability to address the causal relationships between risk factors and diabetes. Secondly, another limitation of this study is that hyperglycemia was diagnosed using a glucometer and capillary blood; it is not as precise and reliable as the estimation of plasma glucose diagnosed using a spectrophotometer/colorimeter.

## Conclusions

From our study on the identification and behavior toward antibiotics of the different germs involved in urinary tract infections in individuals in the Diabetology Unit of Bafoussam Regional Hospital, it emerges that antibiotic resistance rates were higher in diabetics compared to non-diabetics. This study also revealed that there is an association between antibiotic resistance and diabetic status. Research and interventions must be focused on the elderly diabetic population in order to fight against the occurrence of drug-resistant uropathogenes.
